# Antibacterial Activity of some Medicinal Mangroves against Antibiotic Resistant Pathogenic Bacteria

**DOI:** 10.4103/0250-474X.65019

**Published:** 2010

**Authors:** P. D. Abeysinghe

**Affiliations:** Department of Botany, University of Ruhuna, Matara, Sri Lanka

**Keywords:** Antibacterial activity, inhibition, mangroves, soxhlet extraction

## Abstract

The antibacterial activity of the leaves and bark of mangrove plants, *Avicennia marina, A. officinalis, Bruguiera sexangula, Exoecaria agallocha, Lumnitzera racemosa*, and *Rhizophora apiculata* was evaluated against antibiotic resistant pathogenic bacteria, *Staphylococcus aureus* and *Proteus* sp. Soxhlet extracts of petroleum ether, ethyl acetate, ethanol and water were prepared and evaluated the antibacterial activity using agar diffusion method. Most of the plant extracts showed promising antibacterial activity against both bacterial species. However, higher antibacterial activity was observed for *Staphylococcus aureus* than *Proteus* sp. The highest antibacterial activity was shown by ethyl acetate of mature leaf extracts of *E. agallocha* for *Staphylococcus aureus*. All ethyl acetate extracts showed higher inhibition against *S. aureus* while some extracts of chloroform, ethyl acetate and ethanol gave inhibition against *Proteus* sp. None of the petroleum ether and aqueous extracts showed inhibition against *Proteus* sp. All fresh plant materials did also show more antibacterial activity against both bacterial strains than did dried plant extracts. Antibacterial activity of fresh and dried plant materials reduced for both bacterial strains with time after extraction. Since *L. racemosa* and *A. marina* gave the best inhibition for bacterial species, they were used for further investigations. Charcoal treated plant extracts of *L. racemosa* and *A. marina* were able to inhibit both bacterial strains more than those of untreated plant extracts. Phytochemical screening of mature leaf, bark of *L. racemosa* and leaf extracts of *A. marina* has been carried out and revealed that leaf and bark contained alkaloids, steroids, triterpenoids and flavonoids. None of the above extracts indicate the presence of saponins and cardiac glycosides. Separated bands of extracts by TLC analysis showed antibacterial activity against *S. aureus*.

For a long period of time in history, plants have been valuable and indispensable sources of natural products for the health of human beings and they have a great potential for producing new drugs[1–3]. Even today people who live near to the forests use plant products to cure chronic diseases. Tropical and sub-tropical areas of the world are bestowed with abundant flora and herbs which have untapped properties, such as antimicrobial, antiviral and antifungal. According to the World Health Organization, plants are a source of compounds that have the ability to combat disease, antimicrobial, antiviral and antifungal activities[1,4]. In addition, medicinal plants have been used for centuries as remedies for human ailments and diseases because they contain components of therapeutic value[5]. Also they are less toxic to humans and environmentally friendly due to less pollutants produced in production and have minimal health hazards[6].

A large amount of revenue of the world has to pay for the health care. Day by day new dreaded diseases are arising. The rise of antibiotic resistant microorganisms is one of the severe problems in health care systems of the world and infectious diseases are the second most serious cause of death worldwide[1,7]. Therefore, new drugs have to be found, in order to combat such diseases and it is essential to find new compounds that have antimicrobial properties. Concerning the above facts, it is worthwhile to screen plant species which have the above properties to synthesize new drugs[1]. There is a rich species composition and 4000 ha of mangroves are present in Sri Lanka[8] and extracts from different mangrove plants are reported to possess diverse medicinal properties[9].

*Acanthus illicifolius, Avicennia marina and Exoecaria agallocha* showed significant analgesic activity[10,11]. A number of mangroves and associates contain substances which show biological activities such as antiviral, antibacterial and antifungal properties[13–15]. The leaf extracts of *Bruguiera cylindrica* and bark of *Rhizophora mucronata* show antiviral activity against Newcastle disease, vaccinia and hepatitis B viruses. Mangroves are widely used by mangrove dwellers for bush medicine e.g. *A. illicifolius* is used for skin disorders, boils and wounds[12]. Numerous medicines derived from mangroves (ashes or bark infusions) can be applied for skin disorders e.g. *Lumnitzera racemosa* and sores including leprosy. They have been reported to treat different kinds of diseases (headaches, boils, ulcers and diarrhea).

Common uses of mangroves in bush medicine are reviewed by Bandaranayake[12]. A number of mangroves and associates contain poisonous substances, which also show biological activities such as antifungal, antibacterial, antifeedant, molluscicidal, and pesticidal properties[10,14]. Mangrove plants are a rich source of steroids, triterpenes, saponins, flavonoids, alkaloids, tannins[11,13,15]. Extracts from different mangrove plants are reported to possess diverse medicinal properties such as antibacterial, anthelmintic[9]. The screening of plant species for anti-microbial activity in the discovery of new sources of economically valuable materials and metabolites with new therapeutic agents is an important task. As a preliminary study, it has found that aqueous and ethanol extracts of some mangrove species have antimicrobial activities.[16] Therefore, it is possible to control infectious agents using natural products responsible for the inhibitory effect on pathogenic microorganisms using mangrove plant extracts. Expanding the same research (instead of using aqueous and ethanol extracts), the aim of this study was to evaluate the antibacterial activity of medicinal mangrove plants using different solvents: chloroform, ethyl acetate, ethanol and sterilized water in order to get maximum compound(s) from the different plant materials and screening them *in vitro* for antibacterial activity.

## MATERIALS AND METHODS

### Plant materials and bacterial strains:

Fresh immature and mature leaves and bark of *A. marina* (Forssk.) Vierh, *A. officinalis* L., and *B. sexangula* (Lour.) Poir., *E. agallocha* L., *L. racemosa* Willd., and *R. apiculata* Blume were collected from Rekawa lagoon (06°03'N-80°50'E) which is located on the south coast of Sri Lanka. Antibiotic resistant bacterial species of *Staphylococcus* and *Proteus* obtained from the General Hospital Matara, Sri Lanka were used as test bacterial species. Both bacterial cultures were maintained in nutrient agar (peptone 5 g, beef extracts 3 g, NaCl 8 g, agar 18 g, 1000 ml of deionized water) at 4° and cultures were stored in 50% glycerol at –80° in an ultra low refrigerator.

### Extraction by Soxhlet extractor:

Plant materials were washed, air dried and used immediately for the extraction. Three hundred grams of the ground of each plant materials of *A. marina, A. officinalis*, and *B. sexangula, E. agallocha, L. racemosa*, and *R. apiculata* were separately crushed to a powder form using sterilized mortar and pestle. These crushed materials were extracted sequentially into 1500 ml of petroleum ether, chloroform, ethyl acetate, ethanol and sterilized water. Resulting extracts in different solvents were evaporated and concentrated to dryness using the rotary evaporator at 50°. Powder was dissolved in the solvents used for extraction: petroleum ether, chloroform, ethyl acetate, ethanol and sterilized water separately and was stored at 4°. Plant extracts were tested for antibacterial activity against these two bacterial strains by agar diffusion technique.

### Testing of antimicrobial activity for plant extracts:

One hundred microliters (~6×10^6^ CFU/ml) from the over night broth cultures of each bacterial strain was added to Petri dishes (143 mm diameter). About 15 ml of half concentrated melted (at about 50°) nutrient agar was mixed in order to homogenize and allowed to solidify. Wells were made using a cork borer (size-3) on the solidified medium. Prepared wells were filled with 200 µg/ml of the original crude extraction of each extract obtained by the Soxhlet extraction method. All tests were performed in duplicates. The diameters of the inhibition zones were measured and their means were calculated. For control, petroleum ether, chloroform, ethyl acetate, ethanol and water were used instead of plant extracts. *Staphylococcus aureus* was resistant to ceftazidime (15 µg/ml), gentanicin (10 µg/ml) and kanamycin (50 µg/ml) while *Proteus* sp. was resistant to gentamicin (10 µg/ml) and kanamycin (50 µg/ml).

### Antimicrobial activity of fresh and dried extracts of *L. racemosa*:

The above procedure was repeated in order to compare the antimicrobial activity of fresh and dried leaf extracts of chloroform, ethyl acetate, ethanol and aqueous of *L. racemosa* and *A. marina*. Since *L. racemosa* and *A. marina* gave the best inhibition results against tested bacteria, the pattern of antimicrobial activity, with the time after extraction, was tested once a month for plant extracts of *L. racemosa* and *A. marina*.

### Inhibition against charcoal treated and untreated plant extracts:

A small amount of activated charcoal was added into each Soxhlet extract of *L. racemosa* and *A. marina* and incubated at 40° for 10 min and filtered through fluted filter paper. Filtrates were evaporated to 10 ml using a rotary evaporator at 50° and were stored at 4°. Antibacterial activity was tested for plant extracts of charcoal treated and untreated plant extracts. Plates were incubated overnight at room temperature. Inhibitory zone were measured by using a metric ruler in both occasions.

### Preparation of plant extracts for phytochemical screening:

Powdered bark and mature leaves of *L. racemosa* (100 g) was extracted with 300 ml of ethanol in a flask (Soxhlet). After about 1 h of refluxing on a steam bath with occasional swirling, the flask was cooled to room temperature. The mixture was filtered and the crud extract was washed with 50 ml of fresh solvents. Since *A. marina* gave somewhat good results for antibacterial activity for both bacterial species, phytochemical screening tests were done for *A. marina* of different extracts of different solvents. For *A. marina*, 100 g of young leaf were sequentially extracted with 300 ml chloroform, ethyl acetate, ethanol and water in Soxhlet extractor. The total volume of the extract was measured and portions were used in the phytochemical screening for alkaloids, saponins, steroids/triterpenoids, cardiac glycosides and flavonoids[17].

### Separation of active components of extracts by thin layer chromatography (TLC):

Silica coated (G6F_254_, 13% CaSO_4_) plates were air dried at room temperature for one hour and dried at 80° in an oven for 30 min. Extract of mature leaves in chloroform, ethyl acetate, ethanol and the extract of bark in ethyl acetate were separated using thin layer chromatography (TLC). A small amount of each extract (10 µl) was spotted at the bottom of the plate. Then plates were placed in a 50 ml beaker containing pure solvents of hexane, petroleum ether, ethyl acetate and diethyl ether. A combination of hexane and diethyl ether, petroleum ether and ethyl acetate, ethyl acetate and water, hexane and water were used as mobile phase with ratio of 9:1, 8:2, 7:3, 6:4, 5:5, 4:6, 3:7, 2:8 and 1:9. Also ethyl acetate:diethyl ether; 1:1, 9:1, 8:2, 7:3, 6:4, 5:5, 4:6, 3:7, 2:8 and 1:9 were used as mobile phase. Beakers were covered with watch glasses. When the mobile phase reached the top of the plate, plates were removed from the developing chamber and dried at room temperature. Plates were developed using UV, I_2_ vapor, phosphomolybdic acid (PMA) and anisaldehyde spray and were visualized under ultra violet light. Each separated band was scraped into a vial and redissolved in 2 ml of solvent, which was used to extract the plant materials, was filtered and allowed to evaporate the solvent. Antibacterial activity was tested for the above separated fractions against bacterial species by agar diffusion technique.

## RESULTS AND DISCUSSION

Extracts from different mangrove plants are reported to possess diverse medicinal properties[9,11]. For example, *A. illicifolius, A. marina* and *E. agallocha* showed significant analgesic activity[10]. Mangroves and mangrove associates possess novel agrochemical products, compounds of medicinal value, and biologically active compounds[13]. Extracts from different mangrove plants and mangrove associates are active against human and plant pathogens[14]. Seventy five plant extracts from mature leaves and bark of *A. marina, A. officinalis, B. sexangula, E. agallocha, L. racemosa* and *R. apiculata* in petroleum ether, chloroform, ethyl acetate, ethanol and water were used to test the growth of *S. aureus* and *Proteus* sp. Antibacterial assays for plant extracts of mangrove plants and the controls were also carried out. Five different extracts (petroleum ether, chloroform, ethyl acetate, ethanol and sterilized water) exhibited different degree of growth inhibition against bacterial strains.

**Fig. 1:**
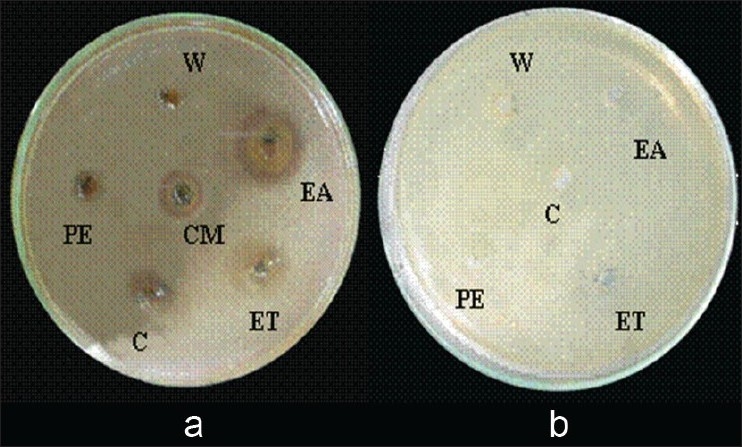
Comparison of growth inhibition of *Staphylococcus aureus* (a) with plant extracts and (b) control. Mature leaves (ML), bark (B) of *L. racemosa* in petroleum ether (PE), chloroform (CM), ethyl acetate (EA), ethanol (ET) and water (W). Inhibition zone was higher for ethyl acetate ethanol and (C) chloroform

**Fig. 2:**
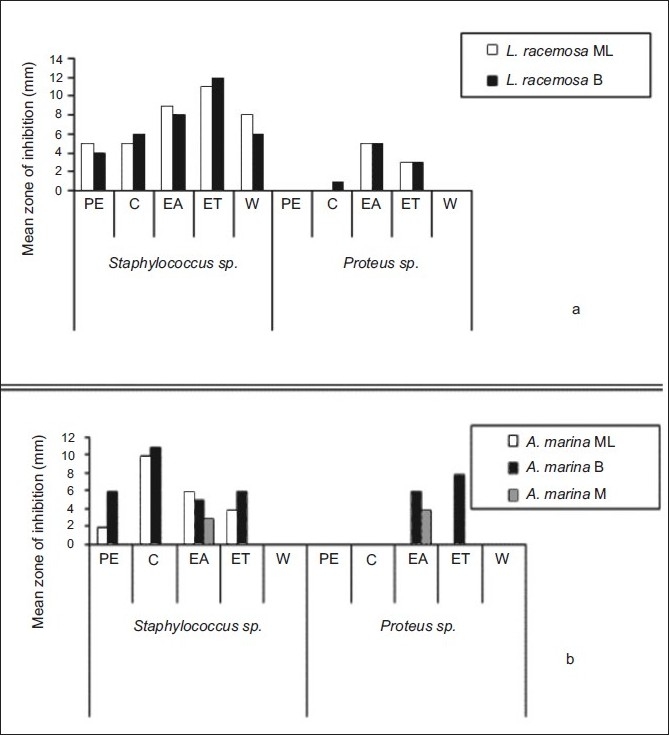
Inhibition of growth of bacteria. Mature leaves (ML), bark (B) of *L. acemosa* and *A. marina* in petroleum ether (PE), chloroform (C), ethyl acetate (EA), ethanol (ET) and water (W)

All plant extracts exhibited more inhibition for *S. aureus* than *Proteus* sp. Almost all plant extract of ethyl acetate showed the highest inhibition compared to the extracts obtained with petroleum ether, chloroform ethanol and water (fig. 1). The highest antibacterial activity for both bacterial strains was shown by plant extracts of *L. racemosa* and *A. marina* among tested mangrove plants (figs. 2a and b). Also, plant extracts of mature leaves and bark of *A. marina, E. agallocha* and *B. sexangula* exhibited considerable inhibition against *S. aureus*.

Out of seventy-five extracts, almost all extracts exhibited the highest antibacterial activity against *Staphylococcus aureus*. Considerable antibacterial activity was shown by ethanol extracts of mature leaves of *E. agallocha* and ethanol extracts of mature leaf and bark of L. recemosa and bark of *L. racemosa* (fig. 2a). Likewise, the highest antibacterial activity was exhibited by ethanol extract of the bark of *A. marina* against *Proteus* sp (fig. 2b). A higher antibacterial activity for both bacterial strains was shown by plant extracts of *A. marina, A. officinalis* and *L. racemosa* among tested mangrove plants. Also, ethyl acetate extracts of mature leaf, immature leaf and bark of *B. sexangula* exhibited antibacterial activity against *Staphylococcus aureus*. In all extracts of ethyl acetate showed the antibacterial activity against *S. aureus* while only a few extracts exhibited the antibacterial activity against *Proteus* sp. This indicates that these plant extracts showed more antibacterial activity against *S. aureus* than *Proteus* sp. None of the petroleum ether and aqueous, mature leaf extract of *A. marina*, immature leaf extracts of *A. officinalis* and *B. sexangula* and mature leaf extracts of *A. marina, E. agallocha*, mature leaf and bark extracts of *R. apiculata* were able to inhibit the growth of *Proteus* sp. but these plant extracts could suppress the growth of *S. aureus*.

According to preliminary study and studies of other people[14], it has been recorded that, a number of mangrove plant extracts of methanol, ethanol and aqueous showed antibacterial activity against pathogenic isolates as well as antibiotic resistant bacteria. Ethanol extracts showed higher inhibition than aqueous extracts. Water extract may contain a low concentration of antibacterial compounds or may not extract antibacterial compound(s). Or all antibacterial compounds may have extracted by other solvents during sequential Soxhlet extraction by petroleum ether, chloroform and ethyl acetate. No inhibitions were given for controls (fig. 1b).

The growth of *S. aureus* was inhibited by most of the plant extracts and some plant extracts inhibited the growth of *Proteus* sp. Therefore, the plant extracts of the above mangrove plant species can be used as a source which could yield drugs to improve the treatment of infection caused by these bacteria. Unlike gram-positive bacteria, the lipopolysaccharide layer along with proteins and phospholipids are the major components of the outer layer of gram-negative bacteria. So the outer lipopolysaccharides layer may hinder access of antibacterial compounds to the peptidoglycan layer of the cell wall. This may be the reason that *Proteus* sp. was resistant to most of the extracts.

Plant extracts of fresh plant materials of *L. racemosa* and *A. marina* could suppress the growth of both bacterial strains more than those of dried plant materials. Inhibition of extracts of fresh plant materials was comparable with the extracts of dried plant materials. Ethanol extract of fresh leaf showed the highest antibacterial activity against *S. aureus*. Ethyl acetate and ethanol extracts of fresh plant materials showed higher antibacterial activity for both bacterial species.

Fresh plant materials may have more antimicrobial compounds than dried tissues and probably during dryness antibacterial compounds may degrade due to temperature. Since fresh materials give much higher antibacterial activities than did dried materials, fresh materials should be used for the extraction in order to obtain maximum antibacterial compounds(s). The degree of growth inhibition of *L. racemosa* and *A. marina* for *S. aureus* reduced slowly with the time after plant extraction until 5^th^ mo. As well as, the antibacterial activity of plant extract reduced very rapidly for *Proteus* sp. with the time after extraction.

**Fig. 3:**
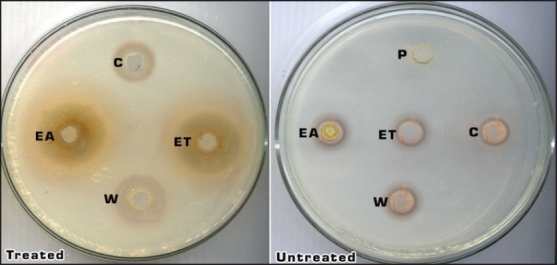
Growth inhibition by charcoal treated and untreated plant extracts. Mature leaves (ML) and bark (B) of L. racemosa in petroleum ether (PE), chloroform (C), Ethyl acetate (EA), Ethanol (ET) and Water (W)

The crude extracts of plant materials contain different types of pigments, such as chlorophyll. To test whether there is an influence of pigments in plant extracts on bacterial growth, charcoal treated and untreated plant extracts were used. A sufficient amount of activated charcoal was used to remove pigments in plant extracts. According to the results given in the fig. 3, charcoal treated plant extracts of *L. racemosa* and *A. marina* were able to inhibit the growth of both bacterial strains more than those of untreated plant extracts (fig. 3). Charcoal treated plant extracts could also inhibit the growth of *Proteus* sp. more than those of untreated plant extracts. It can be assumed that plant pigments may contribute to the increase of bacterial growth and may increase the survival ability of bacteria.

The results of phytochemical screening of ethanol extracts of mature leaf and bark of *L. racemosa* and *A. marina* five extracts young leaf of chloroform, ethyl acetate, ethanol and sterilized water showed the presence of alkaloids, steroids/triterpines and flavonoids. None of the extracts showed positive results for saponins and cardiac glycosides. Positive results resulted from flavonoid tests in all the plant extracts except petroleum ether extract. Alkaloid, flavonoid, tannin and other phytochemicals are present in other plants as well[18]. Phytochemical screening showed that mangroves contain these secondary metabolites which may show antibacterial activity. Therefore, the beneficial medicinal effects of plant materials may result from the combinations of secondary products present in the plant. Secondary products play a role in a plant's defense through cytotoxicity towards microbial pathogens this could prove the usefulness of these as antimicrobial medicines for humans[19]. In addition to above tested mangrove plant species, other mangrove plants can be used to identify and isolate antibacterial components against pathogenic bacterial species.

Hexane, diethyl ether, petroleum ether, ethyl acetate and water and combination of hexane and diethyl ether, petroleum ether and ethyl acetate, ethyl acetate and water, hexane, water and different combinations of solvents were used to carry our TLC. All pure solvents hexane, petroleum ether, ethyl acetate and diethyl ether alone did not give a good separation. When combinations of above solvents used as the mobile phase these did not give successful results either. However, when TLC was carried out, chloroform extracts of mature leaves was separated into two bands (ethyl acetate:diethyl ether 1:1), mature leaves in ethanol were separated into three bands (when ethyl acetate:diethyl 9:1 used) and ethyl acetate extracts of bark separated into two bands (ethyl acetate:diethyl ether 1:1). Separated components in TLC analysis could not inhibit the growth of *Proteus* while *Staphylococcus* sp. was inhibited by some separated fragments.
